# Relationship between Lower Extremity Fitness Levels and Injury Risk among Recreational Alpine Skiers: A Prospective Cohort Study

**DOI:** 10.3390/ijerph191610430

**Published:** 2022-08-22

**Authors:** Zi Wang, Yihui Cai, Junqi Wu, Siyuan Xie, Wei Jiao

**Affiliations:** 1 School of Sports Medicine and Rehabilitation, Beijing Sport University, Beijing 100084, China; 2 School of Strength and Conditioning Training, Beijing Sport University, Beijing 100084, China

**Keywords:** snow sport, physical fitness, injury prevention, risk assessment, logistic regression analysis

## Abstract

Background: Although the importance of physical fitness for injury prevention is recognized in sports medicine and rehabilitation, few studies have investigated this factor among recreational alpine skiers. Objective: To determine the effect of lower extremity fitness on the risk and severity of injury among recreational alpine skiers. Method: This prospective cohort study involved 117 recreational skiers at two alpine resorts during the 2021–2022 winter season. Anthropometric characteristics, skiing skills, and lower extremity agility (hexagon test), balance (Y-Balance Test), and endurance (60-s squat test) were assessed before the winter season. All of the participants were divided into an injured group and an uninjured group, based on whether an injury was recorded throughout the season. Results: In binary logistic regression, the hexagon test duration and composite Y-Balance Test score were significant injury risk factors (*p* < 0.05). Ordinal polytomous logistic regression revealed no significant factors for injury severity (*p* > 0.05). Conclusions: Recreational alpine skiers with inferior lower extremity agility or balance may have a higher injury risk and this must be considered when assessing individual risk. In the context of injury prevention, regular neuromuscular training and testing, including agility and balance aspects should be recommended to skiers.

## 1. Introduction

Alpine skiing is a popular sport worldwide but involves a significant risk of injury [1,2]. Recreational skiers experience 1.9–3.5 incidents per 1000 skiing days [3]. With 400 million skiing days worldwide [4], an estimated 760,000 to 1.4 million injuries may occur annually. Injuries of the lower extremities are the most common alpine skiing-related injuries [2,5,6,7,8,9,10,11], especially to the knee [5,12,13]. Around half of all serious knee injuries are knee sprains, e.g., of the anterior cruciate ligament [14]. Considering the significant contribution of alpine skiing to the total annual number of sports-related injuries [15,16,17], and the social needs of maintaining a physically active lifestyle for people, the injury risk factors and prevention strategies are important areas of concern. The studies performed with skiers found that recent developments in skiing equipment and prevention measures have reduced the frequency and severity of skiing-related injuries. Newly developed bindings are adjusted based on weight, height, age, trail difficulty, and speed [18]. This may account in part for this considerable reduction in lower leg injuries [19]. Some evidence suggests that functional knee bracing seems to reduce the incidence of the anterior cruciate ligament re-injury by providing some degree of stability in the setting of rotational stress in skiers [18,20]. Nevertheless, alpine skiing remains one of the riskiest recreational activities in terms of injury incidence [17,21,22]. So, additional efforts are required to improve the safety of the sport. In addition to extrinsic factors (e.g., protective equipment), the focus of the research has turned to other modifiable intrinsic factors (e.g., physical fitness) that may prevent initial injuries [18]. In theory, the concurrent advancements in sports biomechanics and the understanding of human physiology should translate into targeted exercises for the prevention of sports injuries. The importance of physical fitness in injury prevention is recognized in sports medicine and rehabilitation [23]. For instance, neuromuscular training, which typically consists of exercises aimed at improving physical fitness aspects, such as agility, balance, and endurance [24,25], is considered an important way to prevent musculoskeletal injury in sports. As alpine skiing involves high velocities, great impact forces, uneven ground, and varying snow conditions, it requires high levels of agility, balance, and endurance [23,24,25,26]. Furthermore, postural stability is permanently challenged in skiing, since the ski boots force the skier into a bent-knee position and the skis and boots produce markedly different weight distributions and lever arms [27]. In terms of ski racers, the individuals who had better jump coordination performance and better neuromuscular control were, in general, at a lower injury risk [28,29]. Additionally, several previous studies on skiers reported a greater injury rate in the nondominant leg compared to the dominant leg [30,31,32]. Promsri et al. [33] revealed that when shifting body weight over the nondominant leg and stabilizing this position, the recreational downhill skiers significantly changed the coordination structure and the control of the lifted-leg movements. They suggested that a bilateral lower extremity asymmetry in sensorimotor control could be considered as a potential mechanism for the asymmetry in the risk of lower-limb injuries between the nondominant and dominant legs.

Based on the current findings, recreational alpine skiers may be considered in order to investigate the relationship between the critical lower extremity fitness levels and injury risk; additionally, some specific injury prevention measures can be recommended to skiers if an association is found. However, even though the importance of physical fitness on a high level is crucial in alpine skiing, limited research was performed concerning recreational skiers’ physical fitness and sports injury. Therefore, the aim of the present study was to assess the effects of lower extremity agility, balance, and endurance on the risk and severity of injury among recreational alpine skiers. We hypothesized that better fitness levels would positively influence the injury risk and severity.

## 2. Methods

### 2.1. Study Design

This prospective cohort study was conducted at the Wanlong Ski Resort and the Galaxy Ski Resort in Zhangjiakou, China, during the 2021–2022 winter season. With the assistance of the staff at the two ski resorts, the participants were recruited through the internet and ski resorts. At the beginning of the season (November 2021), all of the participants were assessed using anthropometric measurements, skiing skill questionnaires, and physical fitness tests. The participants’ injuries were recorded throughout the season (November 2021–March 2022). The data were then statistically analyzed.

### 2.2. Participants

A total of 117 recreational alpine skiers were recruited at the two ski resorts in November 2021. Table 1 presents the inclusion and exclusion criteria. The inclusion criteria were as follows: healthy recreational alpine skiers aged 18–40 years; 4–6 h per skiing day; 40–50 guaranteed days of alpine skiing (recorded weekly by telephone in that season) and high physical activity levels achieved (estimated weekly by using the International Physical Activity Questionnaires in that season [34,35,36]) during that winter season; and intermediate or higher skiing skills. The exclusion criteria were as follows: current or recently retired skiing athletes; recent injury requiring medical attention; significant musculoskeletal, neurological, visual, vestibular, cardiorespiratory, or cognitive disorders; pregnancy; and regular participation in other sports or exercises during the winter season. All of the volunteers were screened by two graduate students with skiing experience and high professionalism in sports rehabilitation, who also conducted all of the surveys and measurements.

Written informed consent was obtained from all of the participants. The study was conducted according to the guidelines of the Declaration of Helsinki, and approved by the Experimental Ethics Committee for Sports Science of Beijing Sport University (Approval number: 2022077H).

### 2.3. Data Collection

#### 2.3.1. Anthropometric Characteristics

The participants were asked to provide information on demographic characteristics (age and gender) and medical history, if any. The body weight (in kilograms) and height (in meters) were measured using a bathroom scale and a cloth measuring tape fixed to a wall, respectively. The participants’ body mass index (BMI) was then calculated using the following formula: body weight/body height^2^.

#### 2.3.2. Skiing Skills

Based on the self-reported performance in turns, the skiing skills were classified as beginner (turning technique: plough); intermediate (turning technique: stem turns); good (turning technique: parallel turns); or expert (turning technique: carving/short turns) [37]. To eliminate unnecessary confounders, the beginners were excluded, as they may be at greater risk of injury due to a lack of basic sports-specific instructions and risk awareness [38,39].

#### 2.3.3. Physical Fitness

The Hexagonal Obstacle Test, Lower Quarter Y-Balance Test (YBT), and 90-s Box Jump Field Test are commonly used to assess ski racers’ agility [40,41], balance [42], and endurance [43], respectively. However, given the limited physical capacity of recreational skiers, and for safety reasons, the process of the Hexagonal Obstacle Test and the 90-s Box Jump Field Test, which pose a high injury risk, was changed. Finally, the Hexagon Test (removing the obstacles), YBT, and 60-s Squat Test were used in the testing battery in consultation with five sports medicine experts and five expert skiers and were associated with skiers’ lower extremity fitness levels. The results are shown in Table 2.

*Hexagon Test (removing the obstacles).* The agility was measured using the Hexagon Test, which has excellent test–retest reliability (intraclass correlation coefficients = 0.938–0.924) [44]. A hexagon with 24-inch sides and 120° angles was marked using tape on the floor, with a 12-inch tape strip in the middle to mark the starting position. At the beginning of the test, the participant stood on the tape strip in the middle of the hexagon facing the front line. On the command “go”, they jumped ahead across the line and then back over the same line into the middle of the hexagon. While maintaining a forward-facing position with the feet together, the participant jumped outside the hexagon and back into it. Each participant was required to complete three full revolutions in clockwise direction. A stopwatch was used to record the time of three full revolutions, which was accurate to 0.1s. A total of three times were conducted, with an interval of 1–2 min between each time, and finally the shortest time was recorded [45]. A shorter completion time indicated superior agility [46]. The data were not recorded if the participant failed to completely jump over each line demarcating the hexagon, and they were required to repeat the trial [46].

*YBT.* YBT was performed under the supervision of two of the investigators, following standardized procedures as described in the Functional Movement Systems manual [47]. YBT is a dynamic test performed in the single-leg standing position. It examines how the core and each leg function under body weight loads. Adequate leg muscle strength, flexibility, core control, and proprioception are required to achieve optimal postural stability [47]. This test has been shown to be reliable [48,49]. Before the test, the participants were fully familiarized with the testing procedures and allowed six practice trials with six reaches in each direction [48]. Then, the test was performed by standing on the foot plate in the center of the YBT instrument. The participants were instructed to maintain a single-leg stance while reaching as far as possible with the contralateral leg, and to return to the starting position on the center platform without losing balance. The test required the participant to reach in three directions—anterior (A), posteromedial (PM), and posterolateral (PL)—with three trials allowed for each leg [48]. The difference between the maximum reach distances of two legs in the three directions (the A difference, PM difference, and PL difference) and the lower composite score between the two legs were recorded. A composite score for each leg was derived using the following equation: composite score = [(sum of the greatest reach in each direction)/(3 × limb length)] × 100 [47]. The limb length was measured from the most prominent aspect of the anterior–superior iliac spine to the distal tip of the ipsilateral medial malleolus [50].

*60-s Squat Test.* The lower extremity muscular endurance was measured by 60-s squat test [51,52]. The participants stood with their feet shoulder-width apart. They were instructed to squat until their thighs were horizontal to the ground and then return to the upright position with knees slightly bent. All of them had to perform as many repetitions as they were able to during 60 s. The number of repetitions that the participants could complete in the proper form was recorded, and only one time per person.

#### 2.3.4. Injury Records

An injury was recorded when a skier consulted or was treated by the ski patrol or first aid staff after an accident [11,53]. In terms of severity, an injury resulting in the loss of 1–3 skiing days was considered minimal, an injury resulting in the loss of 4–7 days was considered mild, an injury resulting in the loss of 8–28 days was considered moderate, and an injury resulting in the loss of more than 28 days was considered severe [54]. The participants suffering injuries while skiing contacted the two research assistants either face-to-face or by telephone. The research assistants recorded the occurrence and severity of each injury and offered the participant treatment advice.

### 2.4. Statistical Analysis

The statistical analyses were performed using Microsoft Office Excel 2019 (Microsoft, Redmond, WA, USA) and IBM SPSS Statistics 26.0 (IBM, Armonk, NY, USA). The Shapiro–Wilk test was used to assess data normality. All of the variables were normally distributed (*p* > 0.05).

Depending on whether they suffered an injury during the season, the participants were divided into an injured group and an uninjured group. The age, gender, body weight, body height, BMI, and skiing skills were compared between the two groups using independent-samples *t*-tests for the continuous variables and chi-squared tests for categorical variables, with values of *p* < 0.05 considered statistically significant. The continuous variables were expressed as means ± standard deviations, whereas the categorical variables were expressed as frequencies and percentages.

Concerning the occurrence of injury, two binary logistic regression analyses (dependent variable: injury yes/no) were performed: univariate logistic regression for all of the fitness indicators correlating with the occurrence of injury and multivariate logistic regression for injury risk factors identified as significant in univariate regression. In both of the analyses, the values of *p* < 0.05 were considered statistically significant.

Regarding injury severity, the ordinal polytomous logistic regression was performed (dependent variable: injury severity) to determine the significant risk factors. Values of *p* < 0.05 were considered statistically significant.

The odds ratios (OR) were used to describe the risk of injury. An OR of 1 indicated that a factor had no effect. An OR of >1 indicated that a factor posed a risk of injury. An OR of <1 indicated that a factor acted preventively. A standardized percentile method was used to classify each indicator into three categories: below the 25th percentile = below average; between the 25th and 75th percentiles = average; and above the 75th percentile = above average [55].

## 3. Results

### 3.1. Participants

Skiing injuries were reported by 57 of the 117 participants (48.7%). For the logistic regression analyses, the sample size should be at least about 10 times the number of variables [56]. For the six predictors in this study, the sample of 57 injured participants was deemed acceptable.

The anthropometric characteristics and skiing skills of all of the participants are presented in Table 3. No significant differences were observed between the injured and uninjured groups (*p* > 0.05).

### 3.2. Injury Occurrence

The results of the univariate binary logistic regression analysis of all of the lower extremity fitness variables are presented in Table 4. The hexagon test duration (Wald = 22.061; *p* < 0.001), composite YBT score (Wald = 15.154; *p* = 0.001), and number of squats (Wald = 12.022; *p* = 0.002) correlated with injury occurrence and were included in the multivariate binary logistic regression analysis. The skiers with longer hexagon test durations (OR > 1), lower composite YBT scores (OR < 1) or lower number of squats (OR < 1) were associated with a higher injury risk. The other fitness variables did not correlate with injury occurrence (*p* > 0.05).

The multivariate binary logistic regression analysis of incorporated variables revealed that the hexagon test duration (Wald = 13.151; *p* = 0.001) and composite YBT score (Wald = 6.567; *p* = 0.037) were significant risk factors for injury (Table 5), whereas the number of squats was not (*p* > 0.05). The skiers with longer hexagon test durations (OR > 1) or lower composite YBT scores (OR < 1) were at a higher risk of injury.

### 3.3. Injury Severity

For all of the recorded injuries, most (43.9%) injuries were classified as minimal. The moderate injuries were the fewest (21.1%). No injuries were severe. The ordinal polytomous logistic regression revealed no parameters significantly associated with injury severity (see Table 6).

## 4. Discussion

### 4.1. Agility and Injury Occurrence

Our analysis revealed that the performance on the hexagon test was the most significant injury risk factor. The skiers with greater agility had a lower risk. This suggests that the difficulty of the Hexagonal Obstacle Test can be reduced by removing the obstacles to effectively assess recreational skiers’ risk of injury. This finding is consistent with our postulation and a previous study that demonstrated the beneficial role of improving jump agility in injury prevention among elite youth ski racers [29].

Better performance on the hexagon test has been associated with a faster change-of-direction (COD) speed [57,58]. The hexagon test requires multilateral jumps to six different positions, which requires a COD task that is also part of the modern ski technique [29]. It can be inferred that certain mechanisms that are important for COD performance, such as shorter ground contact times [59], and greater horizontal braking and propulsive forces [60], are also the key mechanical factors for good performance on the hexagon test. A longer ground contact time is associated with increased knee loading, which is a risk factor for knee injury [61]. The COD speed training could be focused on tackling the movement deficits associated with high injury risks [62,63,64] and promoting the techniques required for faster performance [63,64,65,66]. A fast COD speed is imperative in skiing, especially for evading hazards, such as cuts, holes, or varying snow conditions [29].

### 4.2. Balance and Injury Occurrence

Four YBT indicators were used in this study: the anterior; posterolateral, and posteromedial differences; and the composite score. Only the composite score, which is more comprehensive than the other three indicators, was statistically significant, suggesting that the skiers with below-average composite scores had a higher risk of injury than those with average or above-average scores. This indicates that the significant differences in the balance performance between the injured and uninjured groups were not limited to a single direction. These results are consistent with previous findings [29,33,67,68].

Modern ski techniques require one to stabilize in a tuck position with the knees bent and shift one’s body weight to one leg to make a turn. Thus, optimal sensorimotor abilities are needed to obtain the correct feeling for achieving the steep and dangerous inward-leaning angles and carving in the snow [69,70]. Moreover, while executing high-risk technical movements, skiers often face unexpected perturbations due to bad weather, rough terrain, and variable snow conditions. Falls resulting from such situations are the common causes of skiing injuries [71,72]. Therefore, an acute sense of lateral and forward–backward balance is vital for preventing injury [73,74,75]. In the YBT, the participants needed to maintain optimal stability on the front and back sides while standing with one leg bent. Skiers are often in a one-legged holding position when they are about to fall. This is the reason we chose the single-leg balance test, even though skiing involves both legs. It can thus be concluded that the composite YBT score is informative for skiing injury prediction.

### 4.3. Endurance and Injury Occurrence

Given the role of fatigue in on-slope injuries and fatalities [76,77,78], more research into the effects of endurance on alpine snow sport-related injury prevention is needed. This study evaluated lower extremity muscle endurance according to the number of squats per minute. The number of squats significantly correlated with injury occurrence in univariate logistic regression, but not in multivariate regression. This suggests that the effect of endurance on injury occurrence may depend on its possible associations with agility and balance.

Even though, from the perspective of injury prevention, we should focus primarily on the significant risk factors (agility and balance), the impact of endurance should not be overlooked. Although the movement pattern in the 60-s squat test is similar to skiers’ squatting in a tuck position, the test may be too simple (compared to the 90-s Box Jump Field Test) to accurately assess the recreational skiers’ endurance levels. Future studies should consider extending the 60-s squat test to 90 s or using the 90-s Box Jump Field Test to assess the recreational skiers’ endurance levels under the protection of two research assistants. Moreover, recreational alpine skiing is associated with a substantial reduction in glycogen storage and increases in blood lactate levels and muscle damage markers [79,80,81], which can cause fatigue and result in injuries, especially in the lower extremities. Proper endurance training helps the body use glycogen more efficiently, reducing the production of lactate and increasing the circulating anti-inflammatory cytokines [82,83], thus delaying fatigue and preventing injury. This may support a previous study suggesting that lower extremity endurance training may prevent skiing-related injuries [21].

### 4.4. Fitness and Injury Severity

Most of the injuries in this study were classified as minimal or mild, and no significant risk factor was found. A study of youth athletes showed that young ski racers with earlier maturing, a lower core flexion–to–extension strength ratio, a shorter drop jump contact time, and a higher drop jump reactive strength index were likely to miss fewer days of training due to injuries [28]. This suggests that the severity of injury may be reduced by improving physical fitness. However, our results may have been influenced by the relatively few injury occurrences and the relatively short observation period.

### 4.5. Limitations

This study has several limitations that need to be noted. First, the sample was small, which necessarily limited the number of physical fitness indicators that could be included. Second, this study involved recreational alpine skiers aged 18–40 years with intermediate or higher skiing skills. Thus, the results cannot be generalized to beginners, other ages, or other types of skiers (e.g., snowboarders). Finally, the skiing environment experienced by each of the participants during the season may be different. This factor was not considered in the analysis and probably reduced the accuracy of identifying the statistically significant risk factors.

### 4.6. Scope for Further Research

Some directions for future research should be noted. First, studies with larger sample sizes and longer observation periods are needed to detect risk factors of injury occurrence and severity. Second, further investigation of the roles of physical fitness, exercise, and training in reducing the incidence and severity of injuries among recreational skiers is encouraged, given their effectiveness in other sporting activities [23]. Finally, it is necessary to consider the relationship between physical fitness and skiing injuries in relation to different factors (such as different ages, genders, and physical activity levels) using subgroup analyses.

## 5. Conclusions

Recreational alpine skiers with inferior lower extremity agility or balance may have a higher injury risk and must be considered when assessing individual risk. As a consequence, well-developed agility abilities, but also balance abilities, are of utmost importance. However, physical fitness does not seem to significantly affect injury severity.

In the context of injury prevention, regular measurements of the lower extremity fitness parameters are crucial to identify recreational skiers’ risks of injury. Based on these present findings, neuromuscular training that focused on agility and balance aspects should be recommended to recreational skiers. Such training would help skiers avoid injury and the associated reduction in physical activity, and ensure that they can continue to enjoy skiing.

## Figures and Tables

**Table 1:** The inclusion and exclusion criteria of the study.

Parameter	Defined Criteria for the Present Study
Participants	Healthy recreational alpine skiers aged 18–40 years
Skiing hours	4–6 h per skiing day
Skiing days	40–50 skiing days
Physical activity level	High
Skiing skills	Intermediate or higher

**Table 2:** Expert questionnaire for fitness tests (*n* = 10).

Measure	Test	Parameter	Strongly Agree	Agree	Neither Agree nor Disagree	Disagree	Strongly Disagree
Agility	Hexagon Test	Duration of three revolutions around a hexagon (in seconds)	80%	20%	0%	0%	0%
Balance	Y-Balance Test	Lower composite score between the two legs + difference between the maximum reach distances of the two legs in three directions: anterior, posteromedial, and posterolateral (in centimeters)	80%	20%	0%	0%	0%
Endurance	60-s Squat Test	Number of squats	70%	30%	0%	0%	0%

**Table 3:** Characteristics of the participants (Mean ± SD).

Variable	Injured Group (*n* = 57)	Uninjured Group (*n* = 60)	*p*-Value
Age (years)	28.82 ± 4.33	29.30 ± 5.10	0.442
Weight (kg)	69.79 ± 12.53	70.71 ± 12.24	0.689
Height (cm)	171.56 ± 7.56	173.65 ± 6.72	0.117
BMI (kg/m^2^)	23.62 ± 3.51	23.35 ± 3.15	0.654
Sex, *n* (%)			0.119
Male	50 (87.7%)	46 (76.7%)	-
Female	7 (12.3%)	14 (23.3%)	-
Skiing skills, *n* (%)			0.420
Intermediate	41 (71.9%)	39 (65%)	-
Good	16 (28.1%)	21 (35%)	-
Expert	0 (0%)	0 (0%)	-

BMI: body mass index.

**Table 4:** Univariate binary logistic regression results.

Variable	B	Wald	*p*-Value	OR	95% CI	
					Lower	Upper
Hexagon test duration (s)		22.061	<0.001 *			
Medium vs. short	1.288	5.310	0.021	3.624	1.212	10.836
Long vs. short	3.398	21.704	<0.001	29.900	7.159	124.879
Composite YBT score		15.154	0.001 *			
Medium vs. low	−2.001	12.907	<0.001	0.135	0.045	0.403
High vs. low	−2.262	12.528	<0.001	0.104	0.030	0.364
A difference (cm)		2.621	0.270			
Medium vs. small	21.571	<0.001	0.998	2,333,465,790	<0.001	-
Large vs. small	22.484	<0.001	0.998	5,815,714,738	<0.001	-
PL difference (cm)		4.064	0.131			
Medium vs. small	−0.158	0.068	0.794	0.854	0.260	2.800
Large vs. small	0.714	1.157	0.282	2.042	0.556	7.497
PM difference (cm)		1.801	0.406			
Medium vs. small	0.256	0.297	0.586	1.292	0.514	3.252
Large vs. small	−0.331	0.411	0.522	0.718	0.261	1.978
Number of squats		12.022	0.002 *			
Medium vs. low	−0.558	1.298	0.255	0.572	0.219	1.495
High vs. low	−1.846	10.924	0.001	0.158	0.053	0.472

* Statistically significant; included in multivariate logistic regression. YBT: Y-Balance Test; A: anterior; PL: posterolateral; PM: posteromedial.

**Table 5:** Multivariate binary logistic regression results.

Variable	B	Wald	*p*-Value	OR	95% CI	
					Lower	Upper
Hexagon test duration (s)		13.151	0.001 *			
Medium vs. short	0.795	1.639	0.200	2.215	0.656	7.482
Long vs. short	2.751	11.992	0.001	15.661	3.300	74.318
Composite YBT score		6.567	0.037 *			
Medium vs. low	−1.590	6.422	0.011	0.204	0.060	0.697
High vs. low	−1.437	3.991	0.046	0.238	0.058	0.973
Number of squats		3.406	0.182			
Medium vs. low	0.137	0.054	0.816	1.147	0.362	3.632
High vs. low	−0.884	1.653	0.199	0.413	0.107	1.590

* Statistically significant. YBT: Y-Balance Test.

**Table 6:** Ordinal polytomous logistic regression results.

Variable	B	Wald	*p*-Value	OR	95% CI	
					Lower	Upper
Hexagon test duration (s)						
Short	−0.224	0.041	0.839	0.799	−2.376	1.929
Medium	0.188	0.102	0.749	1.207	−0.964	1.340
Long	0	-	-	1	-	-
Composite YBT score						
Low	0.682	0.633	0.426	1.978	−0.999	2.363
Medium	0.214	0.065	0.798	1.239	−1.430	1.859
High	0	-	-	1	-	-
A difference (cm)						
Medium	0.251	0.156	0.693	1.285	−0.995	1.497
Large	0	-	-	1	-	-
PL difference (cm)						
Small	−0.576	0.321	0.571	0.562	−2.572	1.419
Medium	−0.141	0.058	0.810	0.868	−1.295	1.012
Large	0	-	-	1	-	-
PM difference (cm)						
Small	0.162	0.037	0.847	1.176	−1.482	1.805
Medium	0.557	0.533	0.465	1.745	−0.938	2.051
Large	0	0.297	-	1	-	-
Number of squats						
Low	−0.149	0.027	0.870	0.862	−1.932	1.635
Medium	−0.091	0.012	0.913	0.913	−1.719	1.537
High	0	-	-	1	-	-

YBT: Y-Balance Test; A: anterior; PL: posterolateral; PM: posteromedial. No A difference is small in the injured group.

## Data Availability

The data presented in this study are available on request from the corresponding author. The data are not publicly available due to privacy restrictions.
